# Whispers of the Heart: Myocardial Infarction Induced by Beta Agonists

**DOI:** 10.7759/cureus.82855

**Published:** 2025-04-23

**Authors:** Anbhigya Kumar Arya, Saurabh Kumar Singh, Chirag Agarwal, Satyajit Padhiary, Devesh Kumar

**Affiliations:** 1 Cardiology, Vardhman Mahavir Medical College and Safdarjung Hospital, New Delhi, IND; 2 Medicine, Atal Bihari Vajpayee Institute of Medical Sciences and Dr. Ram Manohar Lohia Hospital, New Delhi, IND

**Keywords:** anterior wall acute myocardial infarction, • complex coronary interventions like primary ptca, copd: chronic obstructive pulmonary disease, left ventricle wall motion abnormality, wellens’s syndrome

## Abstract

Wellens syndrome is a relatively common yet fatal presentation of acute coronary syndrome and is considered a ST-segment elevation myocardial infarction (STEMI) equivalent. It often indicates an obstructive lesion in the proximal left anterior descending (LAD) artery and warrants early coronary angiography and revascularisation. We report the case of an elderly female in her 60s who presented to a primary health care centre with an acute-onset shortness of breath. A provisional diagnosis of acute exacerbation of chronic obstructive pulmonary disease (COPD) was made, and the patient was nebulized with salbutamol and cortisone. However, her condition worsened over the next hour, and she was transferred to our hospital. On arrival at our hospital, the patient was hypotensive and extremely distressed. A 12-lead ECG showed STEMI with elevation in leads V1-V6, lead I, and aVL. A diagnosis of anterior wall MI (AWMI) was confirmed. Angiography revealed thrombotic occlusion of the proximal LAD artery. This was subsequently tackled by implanting a drug-eluting Stent. Our patient presented with dyspnoea (an uncommon anginal equivalent) and mild wheezing on auscultation, which initially led to an erroneous diagnosis of acute exacerbation of COPD, as the serum troponins were initially normal and the ECG seemed apparently normal. On retrospective analysis, the clinician had missed a subtle biphasic T wave in V1-V3 with preserved R waves, suggesting Wellens syndrome.

## Introduction

We present a case of ST-elevation myocardial infarction (STEMI) equivalent, which was initially misdiagnosed and erroneously treated, leading to the presentation as frank STEMI. Acute coronary syndrome has been considered a life-threatening condition since time immemorial, particularly when associated with complications. Although mortality rates have come down thanks to timely interventions and availability of in-window percutaneous coronary interventions, in resource-limited nations like ours, timely interventions have not been widely available. Studies have shown an increased risk of acute MI in patients with ischemic heart disease who have been recently started on beta-2 agonists [1,2]. This risk is even higher in patients with chronic obstructive pulmonary disease (COPD) [3,4]. We discuss a case of a patient presenting with a Wellens pattern on ECG, who was initially wrongly diagnosed and later presented with an extensive anterior wall MI (AWMI) [5,6].

## Case presentation

An elderly female in her 60s presented to a primary health care centre with acute-onset shortness of breath for three hours. The patient had no prior history of coronary artery disease, diabetes, or hypertension. She was a chronic tobacco smoker with a smoking index of 250. On examination, she had a heart rate of 110 beats/minute, a respiratory rate of 30/minute, and bilateral rhonchi. She had been provisionally diagnosed with acute exacerbation of COPD and was treated with nebulization with beta agonists and steroids, albeit with minimal relief of symptoms. After two hours of treatment, she had no relief from symptoms and was referred to our emergency department. During transfer, the patient had increased intensity of chest pain with diaphoresis and worsening sensorium. 

On arrival at the emergency department, the patient was diaphoretic, tachycardic with a heart rate of 120/minute and respiratory rate of 40/minute with bilateral basilar crepitations and raised Jugular venous pressure (JVP) and saturation of 90% on room air. Prompt ECG showed ST-segment elevation in leads V2 to V6, suggesting an acute AWMI. A diagnosis of acute AWMI classified as Killip Class II was made. Bedside Transthoracic 2D echocardiography showed a left ventricular ejection fraction of 30% with hypokinesia of the anterior wall, antero-septal wall, and antero-lateral wall [left anterior descending (LAD) territory]. Subsequently, the patient was shifted to the cardiac catheterization laboratory.

**Figure 1:**
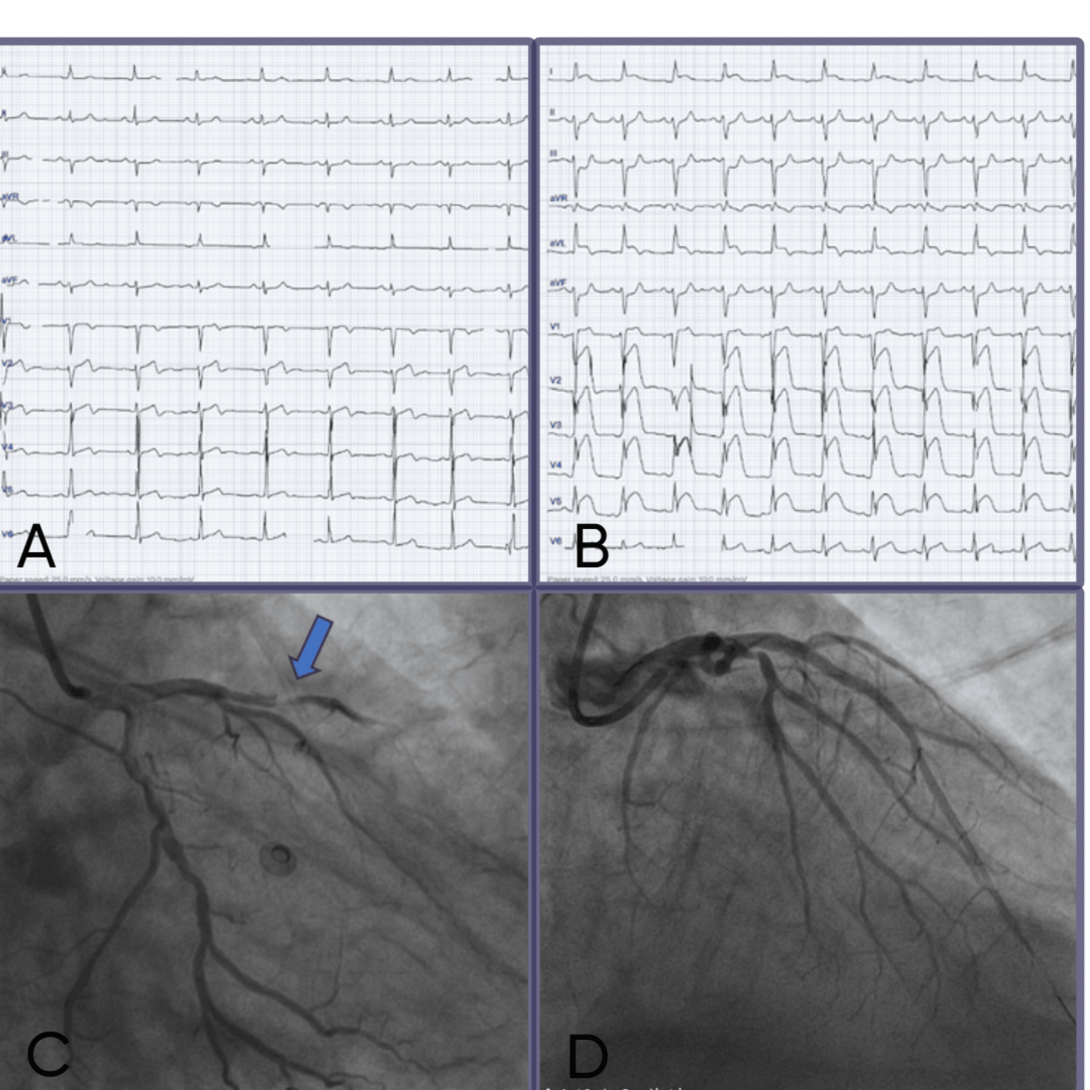
Evolution of ECG changes with angiogram correlation (A) ECG showing subtle biphasic T waves in leads V1-V3 consistent with Wellens type A pattern. (B) ECG showing ST-segment elevation in leads V2 to V6, suggesting an acute anterior wall myocardial infarction. (C) Coronary angiogram in right anterior oblique cranial view showing 95% obstruction in proximal LAD (blue arrow). (D) Post drug-eluting stent implantation, TIMI3 flow was achieved ECG: electrocardiogram; LAD: left anterior descending

Using 7 French right femoral access, the left main coronary artery was hooked with a 6 French Judkins left 3.5 (JL 3.5) catheter. Angiogram revealed 95% obstruction in the proximal to mid LAD artery (Figure 1C). The lesion was crossed with a workhorse (runthrough) wire, and after pre-dilation with a 2.5 x 15 mm semi-compliant balloon, TIMI 2 flow was restored. Slow flow was managed with intra-coronary injections with adenosine 120 micrograms, nitro-glycerine 250 micrograms, adrenaline 10 micrograms, and diltiazem 50 micrograms. A 3 x 32 mm drug-eluting stent (DES) was deployed at 16 atmospheric pressure with the restoration of TIMI 3 flow distally (Figure 1D). The patient was shifted to ICCU for observation and tapered off inotropes. She was subsequently discharged and was doing well at the three-month follow-up.

## Discussion

This was a case of a STEMI equivalent Wellens Syndrome, which was erroneously misdiagnosed as a case of acute exacerbation of COPD. Subsequently, the patient was erroneously treated with beta agonists, culminating in a presentation of frank anterior wall STEMI. Acute coronary syndrome necessitates that physicians be able to timely diagnose and intervene [7]. Studies have shown an increased risk of acute MI in patients with ischemic heart disease who have been recently started on beta-2 agonists [8]. This risk is increased in patients with COPD [2,6]. As seen in our case, the presentation as a STEMI equivalent was missed, and beta-2 agonist inhalation exacerbated the condition to a more acute anterior wall STEMI [3]. This association could be attributed to the stimulation of cardiac beta-2 receptors resulting in tachycardia and leading to increased myocardial oxygen demand and reduced diastolic coronary perfusion. The pathophysiological basis lies in their sympathomimetic properties. Beta agonists increase heart rate, myocardial contractility, and systemic oxygen demand, all of which can unmask or exacerbate underlying coronary insufficiency.

Additionally, beta agonists, especially at high doses, may lead to hypokalemia, arrhythmias, and increased platelet aggregation, further potentiating ischemic events [4,7]. In our case, the presentation with STEMI equivalent was misdiagnosed as an acute exacerbation of COPD. The patient was nebulized with beta agonists, which led to compromised coronary perfusion and tachycardia. Repeated exposure to inhaled beta agonist led to an acute STEMI. Patient-specific factors also play a role in susceptibility to beta agonist-induced cardiac events. These include pre-existing coronary artery disease, advanced age, smoking, hypertension, and electrolyte imbalances. In such populations, even therapeutic doses may pose a risk, particularly during acute exacerbations where the dose or frequency of beta-agonist administration is often increased [3]. A retrospective analysis of the patient’s ECG at first presentation in the primary care hospital revealed a subtle biphasic T wave consistent with Wellens type A pattern on ECG (Figure 1A). We hypothesize that nebulization with beta agonists during stay at the primary care centre would have led to an AWMI and subsequent presentation. 

STEMI equivalent should be suspected when a patient presents with symptoms of angina or angina equivalents - such as chest pain, diaphoresis, or radiation to the jaw or arm - but the ECG lacks classic ST-segment elevations. One should be particularly alert if the ECG shows isolated ST depression in the anterior leads (V1-V3), which may indicate a posterior MI, or if there is diffuse ST depression with ST elevation in aVR or V1, suggesting left main or severe multivessel disease. Tall, symmetric T waves in precordial leads during ongoing chest pain may represent hyperacute changes, while biphasic or deeply inverted T waves in V2-V4 during pain-free intervals point toward Wellens syndrome and critical proximal LAD stenosis.

Another key pattern is De Winter’s, which presents with upsloping ST depression and prominent T waves in precordial leads, often indicating proximal LAD occlusion. A new or presumed new left bundle branch block or paced rhythm in the setting of ischemic symptoms, especially with hemodynamic instability, should also raise concern, and the use of the modified Sgarbossa criteria should be considered. Additionally, STEMI equivalents should be on physicians' radar when encountering patients with ongoing chest pain despite medical therapy, rising troponins with non-diagnostic ECGs, or clinical instability such as hypotension or arrhythmias without clear ECG evidence of STEMI. In all these cases, early recognition and emergent reperfusion are critical.

## Conclusions

Beta agonists are a vital weapon in the therapeutic armamentarium for the management of COPD and its acute exacerbations, but they can act as a double-edged sword in patients with underlying risk factors for cardiovascular disease. Providers should be aware of this diagnosis, and early referral for patients presenting with such symptoms is key. Early referral for patients who fail to improve at the primary care centre will also help in reducing the overall morbidity and mortality associated with MI. Also, recognizing STEMI equivalents requires a high index of suspicion, especially when classic ECG criteria for STEMI are absent in patients with ongoing ischemic symptoms or clinical instability. Assessment of the clinical picture, along with careful ECG interpretation and awareness of high-risk patterns, is essential to avoid delays in reperfusion therapy. Early identification and appropriate action can significantly improve outcomes in patients with acute coronary occlusion presenting with these atypical but life-threatening ECG patterns.
